# Study on Removal Mechanism for Copper Cyanide Complex Ions in Water: Ion Species Differences and Evolution Process

**DOI:** 10.3390/ijms24065066

**Published:** 2023-03-07

**Authors:** Ying Liu, Baogang Sun, Wenting Jia, Yuan Wang, Lijia Huang, Pengge Ning, Shaojun Yuan

**Affiliations:** 1 Low-Carbon Technology and Chemical Reaction Engineering Lab, School of Chemical Engineering, Sichuan University, Chengdu 610065, China; 2 Chemistry and Chemical Engineering Data Center, Institute of Process Engineering, Chinese Academy of Sciences (CAS), Beijing 100190, China

**Keywords:** wastewater treatment, ion species, cyanide, deep removal, complexation mechanism, cupric ion

## Abstract

A large amount of cyanide-containing wastewater is discharged during electrode material synthesis. Among them, cyanides will form metal–cyanide complex ions which possess high stability, making it challenging to separate them from these wastewaters. Therefore, it is imperative to understand the complexation mechanism of cyanide ions and heavy metal ions from wastewater in order to obtain a deep insight into the process of cyanide removal. This study employs Density Functional Theory (DFT) calculations to reveal the complexation mechanism of metal–cyanide complex ions formed by the interaction of Cu^+^ and CN^−^ in copper cyanide systems and its transformation patterns. Quantum chemical calculations show that the precipitation properties of Cu(CN)_4_^3−^ can assist in the removal of CN^−^. Therefore, transferring other metal–cyanide complex ions to Cu(CN)_4_^3−^ can achieve deep removal. OLI studio 11.0 analyzed the optimal process parameters of Cu(CN)_4_^3−^ under different conditions and determined the optimal process parameters of the removal depth of CN^−^. This work has the potential to contribute to the future preparation of related materials such as CN^−^ removal adsorbents and catalysts and provide theoretical foundations for the development of more efficient, stable, and environmentally friendly next-generation energy storage electrode materials.

## 1. Introduction

Electrochemical energy storage (EES) technology has received a lot of attention in recent years due to the increasing demand for sustainable and efficient energy storage solutions around the world [1,2]. Therefore, EES technology is widely used in various fields, including electric vehicles, clean energy storage, portable electronic products, and aerospace [3,4,5]. Among them, electrode materials, as key components of EES devices, have received extensive attention [6]. They play a crucial role in the storage and release of electrical energy, directly impacting the overall performance and efficiency of EES devices [7]. Due to the increasing demand for clean energy and efficient energy storage solutions, the demand for high-performance EES devices continues to grow [8,9,10]. Therefore, the development and commercialization of advanced electrode materials have become the main focus of industrial and research efforts, and more and more factories and laboratories are beginning to develop and prepare electrode materials on a large scale [11,12,13]. Despite the promising commercial prospects of electrode materials, there are also environmental and health issues related to their production and use. For example, precursors and reagents containing cyanides are used in electrode synthesis, and some synthesis methods even adopt cyanide electroplating processes, resulting in the inevitable production of large amounts of cyanide-containing wastewater [14,15,16]. In addition, heavy metal ions such as Cu^2+^, Al^3+^, Mn^2+^, Cr^3+^, and Ni^2+^ are also released into the wastewater [17,18,19]. Cyanide is highly toxic and can cause serious health problems if ingested or inhaled. It can also cause harm to aquatic life and have serious negative impacts on ecosystems if released into water bodies [20]. Therefore, the presence of cyanide in wastewater can pose significant environmental and health hazards [21]. Currently, there are about three ways to remove cyanide from this wastewater, including physical, chemical, and biological treatment methods. Physical treatment methods involve removing cyanide from wastewater through physical processes such as precipitation, filtration, and evaporation [22,23]. Chemical treatment methods involve the use of chemical reactions to convert cyanide into less toxic compounds [24,25]. Biological treatment methods involve the use of microorganisms, such as bacteria and fungi, to break down cyanide into non-toxic compounds [26,27,28]. However, these conventional methods are often unable to effectively remove all cyanides, as cyanides in wastewater form stable complexes that are resistant to destruction by traditional removal techniques, thus hindering the achievement of deep removal.

At present, many researchers have inferred through experiments that cyanide ions may form metal–cyanide complexes by bonding with metals [29,30]. Routledge et al. [31] discovered that cyano ions interact with lanthanide element complexes in water and bind with lanthanide elements as a ligand to form new complexes, thereby replacing the original ligand in the lanthanide element complex. Chen et al. [32] found that Ir, Pd, and Pt form complexes with cyanides and determined the structures of the related complexes through characterization methods. Kurashova et al. [33] found that cyanides form related complexes with Fe^3+^ and studied the reaction kinetics of Fe^3+^ forming complexes with cyanides. Andrews et al. [34] discovered that Zn, Ge, and Hg react with cyanides to form complexes and determined the related structures of cyanide complexes and isocyanide complexes through experimental characterization. Therefore, it is necessary to delve into the binding mechanism of cyano ions and metals to facilitate the removal of metal–cyanide complexes in the future. Castilla-Acevedo et al. discovered that Co^3+^ forms complexes with CN^−^ and found in the study that the degradation of strong cyanide complexes could be achieved by using UVC activation of PS to study the mechanism of degradation by activation. Al-Jibori et al. [35] studied the reaction process of Pd (II) forming metal–cyanide complexes with cyanides and discovered that Pd (II) complexes may have a binding mechanism that enhances the bonding process as an oxidation catalyst. Xu et al. [36] studied the binding mechanism of Cu(II)-EDTA by UV/persulfate and UV/H_2_O_2_ [37].

In this work, we studied the complexation mechanism of Cu^+^ and CN^−^ combined to form metal–cyanide complex ions, studied the thermodynamic process of metal–cyanide complex ions in solution, and clarified the transformation law and transformation path between metal–cyanide complex ions. It is clear that Cu(CN)_3_^2−^ is the enriched complex ion in solution, and Cu(CN)_4_^3−^ is the key dominant ion form for the conversion of complex ions to precipitation in the dominant solution to achieve deep removal. The optimum process parameters of Cu(CN)_4_^3−^ under different conditions were analyzed and determined to maximize the depth of the removal of CN^−^ from the solution. This work is expected to provide a solid theoretical foundation for the development of new-generation electrode materials for batteries and supercapacitors and offer theoretical guidance for the development of efficient, energy-saving, and environmentally friendly cyanide removal processes.

## 2. Results and Discussion

### 2.1. Geometrical Configuration of Copper Cyanide Complex Ions

In copper cyanide solution, due to the abundant existence of CN^−^, CN^−^ will reduce Cu^2+^ to Cu^+^. Cu^+^ has a strong coordination ability in the solution and is easily combined with anions in the solution to form stable metal complex ions.

Theoretically, Cu can exist in both four-coordination and six-coordination forms. However, the coordination between Cu and CN^−^ in real systems is unknown. To clarify the ionic form of copper cyanide solutions and achieve deep removal of CN^−^ and metal ions, a database screening of copper cyanide solutions was performed. Firstly, the SC database was used to screen metal–cyanide complexes with CN as the ligand, resulting in 76 metal complexes that can coordinate with cyanide. Secondly, the Crystallography Open Database (COD) and Cambridge Structural Database (CSD) were used to screen for other anionic ligands forming relevant products with Cu(I) as the central metal ion. The screening results showed that most of the Cu(I) in the solution combined with anions in four-coordination forms. Therefore, it is suggested that Cu(I) in solution is more inclined to form related complexes in the four-coordination form.

**Figure 1:**
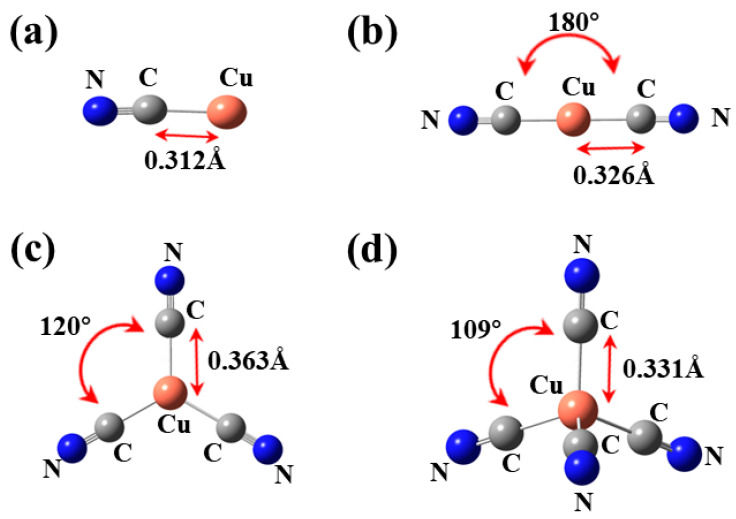
Optimized Geometry Structures of (**a**) CuCN, (**b**) Cu(CN)_2_^−^, (**c**) Cu(CN)_3_^2−^, and (**d**) Cu(CN)_4_^3−^.

The structures of all ionic states (CuCN, Cu(CN)_2_^−^, Cu(CN)_3_^2−^ and Cu(CN)_4_^3−^) involving Cu(I) in a cyanide solution were optimized, and the optimized structures are depicted in Figure 1. The optimized key length information is shown in Table S1.

As depicted in Figure 1, CuCN adopts a linear structure, while Cu(CN)_2_^−^ exhibits a symmetrical linear structure with a bond length of 0.326 Å and a bond angle of 120° between the central atom Cu and CN. Cu(CN)_3_^2−^ exhibits a stable triangular geometry after optimization, with a bond length of 0.363 Å and a bond angle of 120° between the central Cu atom and CN. Cu(CN)_4_^3−^ adopts a stable tetrahedral geometry after optimization, with a bond length of 0.331 Å and a bond angle of 109° between the central Cu aton and CN. By comparing the optimized bond length and bond angle of the three complex ions in the solution, it was determined that the bond length of Cu(CN)_3_^2−^ (0.363 Å) > Cu(CN)_4_^3−^ (0.331 Å) > Cu(CN)_2_^−^ (0.326 Å). According to the principle that a shorter bond length results in higher energy and greater stability, the conclusion was drawn that the stability in the solution is in the order of Cu(CN)_2_^−^ > Cu(CN)_4_^3−^ > Cu(CN)_3_^2−^ based on the calculated bond lengths. This conclusion contradicts the results reported in the literature [38]; therefore, it is necessary to investigate the stability of each complex ion in the system and the reasons for this stability. Based on the bond angle theory, it is speculated that bond angles in this system may have a greater impact on the structure than bond lengths. As bond angles become larger, the repulsion between bonding electrons becomes smaller and more stable. Therefore, in subsequent studies, we focus on exploring the copper cyanide chelate ions formed by Cu^+^ and CN^−^.

### 2.2. Electrostatic Potential (ESP) Analysis

**Figure 2:**
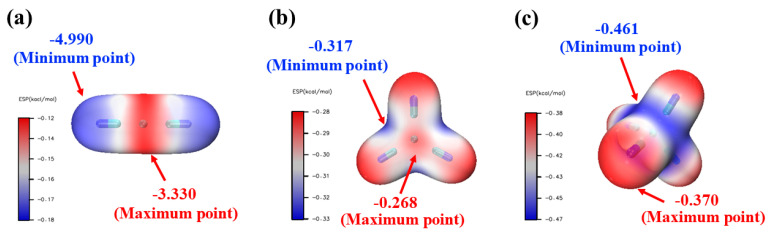
ESP isosurfaces for (**a**) Cu(CN)_2_^−^, (**b**) Cu(CN)_3_^2−^, and (**c**) Cu(CN)_4_^3−^.

ESP (Electrostatic Potential) refers to the interaction energy between the molecular charge distribution and a unit positive charge; it is typically studied on the VDW surface (an isosurface of electron density (q) equal to 0.001 a.u.). Figure 2 displays the VDW surface and surface extrema of the ESP mapping, where red and blue denote positive and negative potential regions, respectively. It is apparent that the C-N fragment of Cu(CN)_2_^−^ is electron-rich and the minimum ESP value of (−4.99 kcal/mol) is located near the N atoms at both ends, while the maximum ESP value is located at the central Cu, indicating a strong reactivity of this fragment with metal ions. Furthermore, the concentration of negative potential regions of Cu(CN)_3_^2−^ and Cu(CN)_4_^3−^ is far greater than that of Cu(CN)_2_^−^, with minimum ESP values of −0.317 kcal/mol and −0.461 kcal/mol for Cu(CN)_3_^2−^ and Cu(CN)_4_^3−^, respectively. However, the difference between their maximum and minimum values is slightly lower than that of Cu(CN)_3_^2−^.Thus, it can be concluded that the stability of the three metal–cyanide complex ions in solution follows the order of Cu(CN)_3_^2−^ > Cu(CN)_4_^3−^ > Cu(CN)_2_^−^. This conclusion corresponds with the stability of the ions in the solution inferred based on the optimized structural information [39,40].

Based on the structural optimization and ESP analysis of the three structures, the state, binding mode, electrostatic potential and stability of the three metal–cyanide complex ions in solution have been determined. Subsequently, the thermodynamics of the system were calculated to further explain the preferred ionic form in solution and to predict their behavior under thermodynamic conditions.

### 2.3. Interaction Region Indicator Analysis

The interaction region indicator (IRI) is essentially a gradient norm of the electron density weighted proportionally by the electron density. The IRI can effectively reveal chemical bonds and weak interaction regions, bringing great convenience to the study of various chemical systems and chemical reactions. The definition of the IRI function is:

(1) $$\mathrm{IRI}\left(r\right)=\frac{\left|\nabla \rho \left(r\right)\right|}{{\left[\rho \left(r\right)\right]}^{a}}$$

where “*a*” is the adjustable parameter and “*ρ*” represents the electron density.

**Figure 3:**
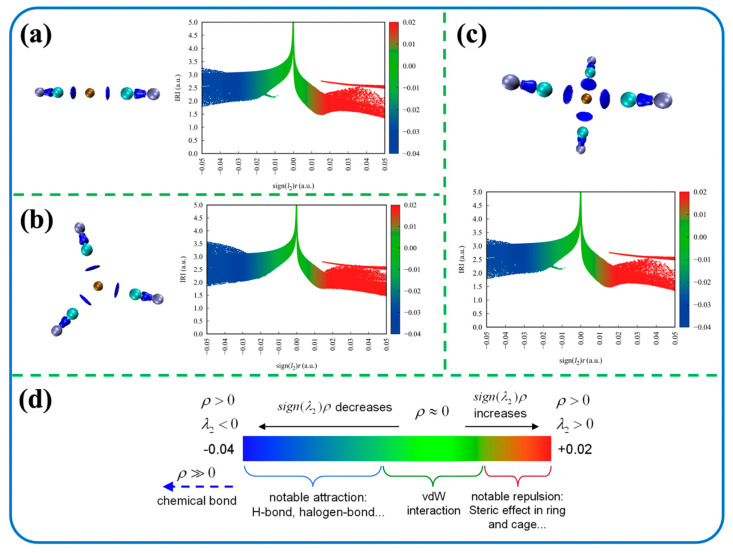
The interaction force structure and scatter diagram of (**a**) Cu(CN)_2_^−^, (**b**) Cu(CN)_3_^2−^, (**c**) Cu(CN)_4_^3−^ and (**d**) coloring method of sign(λ_2_)ρ.

In the above discussion, thermodynamic calculations were employed to determine that the enriched ionic form in the solution was Cu(CN)_3_^2−^ and the predominant ionic form in the solution was Cu(CN)_4_^3−^. In order to further elaborate on the bonding mechanism, the IRI intermolecular force analysis was performed on Cu(CN)_2_^−^, Cu(CN)_3_^2−^ and Cu(CN)_4_^3−^ complexes of metal–cyanide complexes, and the resulting intermolecular force structures and scatter plots of intermolecular forces for each ion are shown in Figure 3. The continuous isosurface maps with a generally green color were found in the overlapping regions between several cyanide ligands (Figure 3a–c). This represents a low electron density in the region, with the intermolecular interaction dominated by P–P overlap effect due to dispersion, since dominant electrostatic interactions usually correspond to high electron densities and should appear as blue. It can also be observed from the structural diagram that the main interaction between the central metal ion Cu and CN^−^ is a chemical bond, while the interaction between the cyanide ion and the solvent water is primarily dominated by van der Waals forces. The original scatter plots of high-resolution electron density distribution for Cu(CN)_2_^−^, Cu(CN)_3_^2−^ and Cu(CN)_4_^3−^ are shown in Figure S1.

### 2.4. Thermodynamic Calculations

Through the calculation of the Gibbs free energy of the reactions involved in the system and the binding energy of metal–cyanide complex ions, further judgment is made on the stability of ions in the system and the conversion paths and patterns between the ions.

The structural optimization of Cu(CN)_2_^−^, Cu(CN)_3_^2−^, and Cu(CN)_4_^3−^ was carried out at the PBE0-GD3/genecp level with an implicit solvent model and dispersion correction, and the single point energy of the molecular/ionic species involved in the copper cyanide system was calculated at the M062x/def2tzvp level. It can be observed that as the number of CN^−^ coordination increases, the single point energy of the metal–cyanide complex ions formed by the combination of Cu and CN^−^ also increases gradually. Finally, the Cu coordination reaches saturation (full coordination number) with Cu(CN)_4_^3−^, and its energy reaches the highest value of 564 KJ/mol.

**Figure 4:**
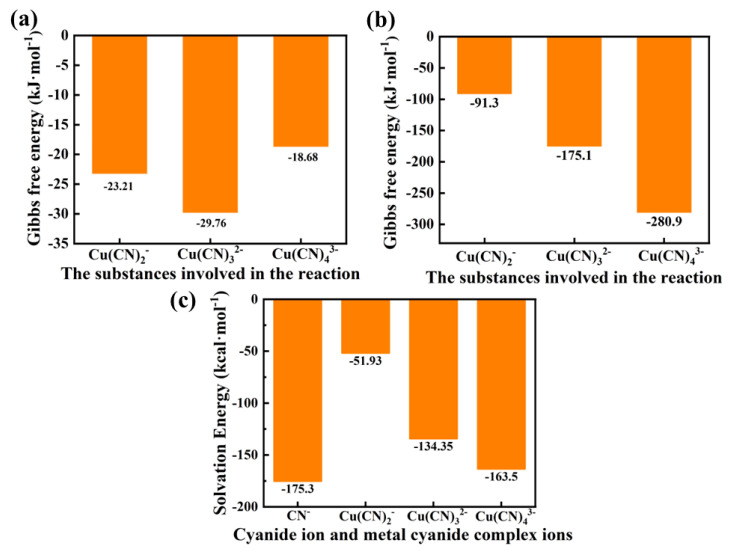
(**a**) The Gibbs free energy (ΔG) of the chemical reactions involving Cu(CN)_2_^−^, Cu(CN)_3_^2−^ and Cu(CN)_4_^3−^. (**b**) The Gibbs free energy (ΔG) of the precipitation reactions involving Cu(CN)_2_^−^, Cu(CN)_3_^2−^ and Cu(CN)_4_^3−^. (**c**) The solvation free energy of CN^−^, Cu(CN)_2_^−^, Cu(CN)_3_^2−^ and Cu(CN)_4_^3−^.

The Gibbs free energy of each substance in the system was calculated, and Equations (2)–(4) were proposed for the various metal–cyanide complex ion forms in the solution. The Gibbs free energy of the reaction was also calculated, as shown in Figure 4b. Relevant thermodynamic data are shown in Table S2. As observed from Figure 4b, the ΔG of the three reactions are all less than 0, indicating that reactions from Equations (2)–(4) in the solution can all proceed spontaneously. This also confirms the presence of Cu(CN)_2_^−^, Cu(CN)_3_^2−^, and Cu(CN)_4_^3−^ in actual solutions. It was found that the Gibbs free energy of the two reactions generating Cu(CN)_2_^−^ and Cu(CN)_3_^2−^ continuously decreases, indicating that the degree of spontaneous reaction is constantly increasing throughout the reaction. However, when Cu(CN)_3_^2−^ transforms into Cu(CN)_4_^3−^, it was found that ΔG was not a decreasing trend, but instead increased, indicating that there was an energy barrier in the transformation of Cu(CN)_3_^2−^ into Cu(CN)_4_^3−^ and the degree of spontaneous reaction was insufficient compared to the previous reaction. By comparing the ΔG of the three reactions, it was found that the reaction generating Cu(CN)_3_^2−^ had the highest degree of spontaneity (minimum ΔG) and the system was more inclined to form Cu(CN)_3_^2−^ because of the energy barrier in the transformation of Cu(CN)_3_^2−^ into Cu(CN)_4_^3−^. Therefore, it is concluded that, in solutions, due to the stable planar triangular spatial structure of Cu(CN)_3_^2−^ and its highest degree of spontaneous reaction in solutions, Cu(CN)_3_^2−^ exists in large quantities in solutions as the enriched ion form. This conclusion is consistent with the related conclusions drawn by researchers in the literature through experiments [41].

(2) $${\mathrm{CuCN}+\mathrm{CN}}^{-}{=\mathrm{Cu}(\mathrm{CN})}_{2}^{-}$$

(3) $${\mathrm{Cu}(\mathrm{CN})}_{2}^{-}{+\mathrm{CN}}^{-}{=\mathrm{Cu}(\mathrm{CN})}_{3}^{2-}$$

(4) $${\mathrm{Cu}(\mathrm{CN})}_{3}^{2-}{+\mathrm{CN}}^{-}{=\mathrm{Cu}(\mathrm{CN})}_{4}^{3-}$$

The thermodynamic calculations of the substances and reactions involved in the system were carried out to clearly determine that Cu(CN)_3_^2−^ is the enriched ionic form in the system and to explain the mechanism behind its formation. Since the majority of CN^−^ in the solution is combined with metal ions to form stable metal–cyanide complex ions, these complex ions are difficult to directly remove from the solution, leading to the inability to achieve deep removal of cyanate in sodium-ion battery discharge wastewater. In order to achieve the deep removal of cyanide from sodium-ion battery discharge wastewater, it is necessary to regulate the formation of CuCN precipitates between Cu and CN^−^ in solution, rather than to form complex ions that cannot be directly removed from the solution, to achieve the deep removal of CN^−^. We need to destroy the enriched form of complex ions in the solution, break the reaction balance and destroy its stable structure so that the CN^−^ in the solution can be transferred to the complex ions conducive to precipitation and so the CN^−^ in the solution can be removed by precipitation to the maximum extent. Therefore, it is necessary to investigate the conversion paths and rules between Cu(CN)_2_^−^, Cu(CN)_3_^2−^, Cu(CN)_4_^3−^, and CuCN(s) in solution.

The Equations (5)–(7) for the formation of precipitation of Cu(CN)_2_^−^, Cu(CN)_3_^2^, and Cu(CN)_4_^3−^ are listed below, and their ΔG results are calculated at the M062x/def2tzvp level, as shown Figure 4c. It can be seen that, as the number of cyanide ligands increases (four ligands reach saturation), the ΔG of CuCN precipitation decreases, indicating that the degree of spontaneous reaction of the reaction increases as the number of cyanide ligands increases. Furthermore, Equation (7) is the most favorable reaction in the entire system to produce precipitation. The solvation free energy of Cu(CN)_2_^−^, Cu(CN)_3_^2−^ and Cu(CN)_4_^3−^ was also calculated, as shown in Figure 4d. It can be seen that the solvation free energy of the three ions decreases as the coordination number increases, and the solvation free energy of Cu(CN)_4_^3−^ is only second to that of free CN^−^. This indicates that Cu(CN)_4_^3−^ has high precipitation characteristics compared to other complex ions and is the dominant ion form in the solution (the dominant ion form refers to the metal–cyanide complex ion form that is most favorable for forming precipitation from the system during the entire reaction process).

(5) $${\mathrm{Cu}(\mathrm{CN})}_{2}^{-}{+\mathrm{Cu}}^{+}=2\mathrm{CuCN}$$

(6) $${\mathrm{Cu}(\mathrm{CN})}_{3}^{2-}{+2\mathrm{Cu}}^{+}=3\mathrm{CuCN}$$

(7) $${\mathrm{Cu}(\mathrm{CN})}_{4}^{3-}{+3\mathrm{Cu}}^{+}=4\mathrm{CuCN}$$

### 2.5. The OLI Simulation Predicts the Distribution of Species in an Actual Solution Environment

In order to further validate the results of the aforementioned simulation calculations, the conversion patterns and transition paths between Cu(CN)_2_^−^, Cu(CN)_3_^2−^, and Cu(CN)_4_^3−^ within the microcopper cyanide system in an actual solution environment were explored using OLI studio 11.0. In order to simulate the actual solution environment more realistically, the initial concentrations simulated were the actual measured concentrations after one treatment of sodium ion battery discharge wastewater.

**Figure 5:**
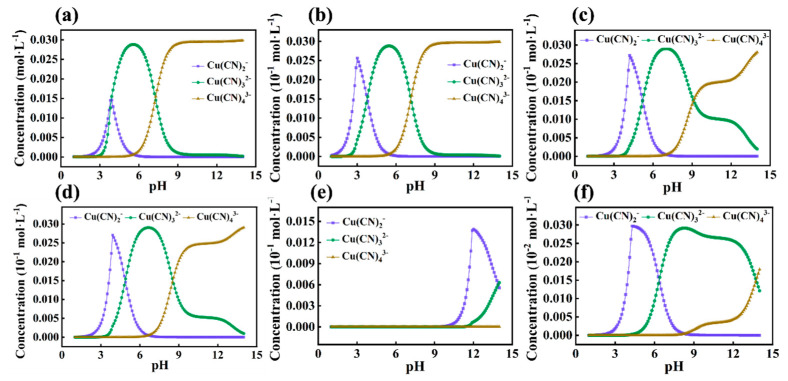
The OLI studio 11.0 was utilized to simulate the distribution of Cu(CN)_2_^−^, Cu(CN)_3_^2−^ and Cu(CN)_4_^3−^ as a function of pH under six different concentration gradients, with the following concentrations: (**a**) c(Cu):c(CN) = 0.03:0.3, (**b**) c(Cu):c(CN) = 0.003:0.3, (**c**) c(Cu):c(CN) = 0.003:0.06, (**d**) c(Cu):c(CN) = 0.003:0.03, (**e**) c(Cu):c(CN) = 0.003:0.003, (**f**) c(Cu):c(CN) = 0.0003:0.003.

Initially, the OLI studio 11.0 was utilized to simulate the morphological distribution of Cu(CN)_2_^−^, Cu(CN)_3_^2−^ and Cu(CN)_4_^3−^ within the system with respect to pH changes under six different concentration gradients. The results are shown in Figure 5. As can be seen from the simulation results in Figure 5a–d, the trends of the changes in Cu(CN)_2_^−^, Cu(CN)_3_^2−^, and Cu(CN)_4_^3−^ in the system are roughly the same when the concentration ratio c(Cu):c(CN) changes from 1:10 to 1:20 and even to 1:100. As the pH continues to increase, Cu(CN)_2_^−^ is rapidly produced in the system, reaching a maximum at pH 3–4 (the region where the maximum appears is positively correlated with the concentrations of various substances in the system). Then, Cu(CN)_2_^−^ rapidly decreases as Cu(CN)_3_^2−^ is generated. Here, a rapid conversion from Cu(CN)_2_^−^ to Cu(CN)_3_^2−^ is achieved. The concentration of Cu(CN)_3_^2−^ then grows exponentially and reaches a peak maximum at pH 6–7 (the region where the maximum appears shifts to the right as the concentrations of various substances in the system decrease). Cu(CN)_4_^3−^ starts to appear in the system at pH 6–7, when Cu(CN)_2_^−^ has completely disappeared and is considered to have been completely converted to Cu(CN)_3_^2−^ and Cu(CN)_4_^3−^ and mostly converted to Cu(CN)_3_^2−^, as can be inferred from Figure 5a–d. As the pH in the system continues to increase, Cu(CN)_3_^2−^ begins to gradually convert to Cu(CN)_4_^3−^ and reaches a reaction equilibrium at pH 9, when Cu(CN)_2_^−^ and Cu(CN)_3_^2−^ have basically disappeared and are completely converted to Cu(CN)_4_^3−^. The amount of precipitated CuCN in the system also reaches its maximum value at this time. This phenomenon directly demonstrates that Cu(CN)_4_^3−^ is the dominant ionic form in the system and that the precipitation of CuCN can be maximized by transferring other metal–cyanide complex ions to Cu(CN)_4_^3−^ through the regulation of the system, thus achieving deep removal of cyanides from wastewater. As can be seen from Figure 5e,f, when the concentration is minimal, only a small amount of Cu(CN)_2_^−^ and Cu(CN)_3_^2−^ are generated within the system. At this time, it is believed that the amount of cyanide ions in the solution is too small to support the transformation of Cu(CN)_3_^2−^ to Cu(CN)_4_^3−^.

**Figure 6:**
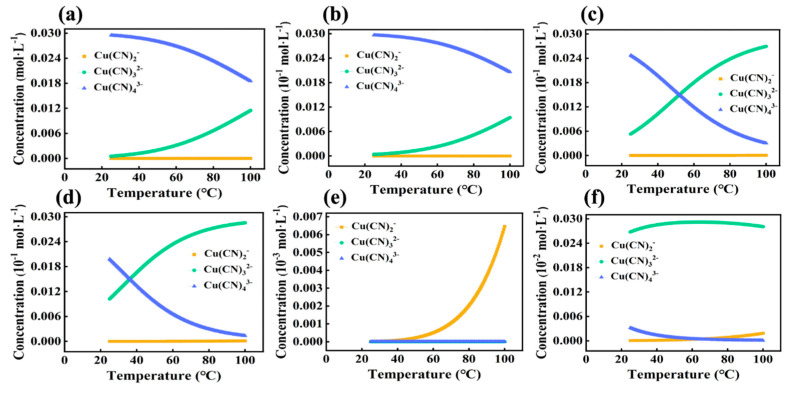
The OLI studio 11.0 was utilized to simulate the distribution of Cu(CN)_2_^−^, Cu(CN)_3_^2−^, and Cu(CN)_4_^3−^ as a function of temperature under six different concentration gradients, with the following concentrations: (**a**) c(Cu):c(CN) = 0.03:0.3, (**b**) c(Cu):c(CN) = 0.003:0.3, (**c**) c(Cu):c(CN) = 0.003:0.06, (**d**) c(Cu):c(CN) = 0.003:0.03, (**e**) c(Cu):c(CN) = 0.003:0.003, (**f**) c(Cu):c(CN) = 0.0003:0.003.

Subsequently, the distribution of Cu(CN)_2_^−^, Cu(CN)_3_^2−^, and Cu(CN)_4_^3−^ in the system under different concentration gradients with temperature changes was explored. The results are shown in Figure 6. It was observed that in cases of excessive CN^−^, the distribution patterns of various ionic forms in the system were generally similar. The amount of Cu(CN)_4_^3−^ decreases gradually with the rise in temperature, and the rate of decrease is faster when the concentration is lower. As temperature increases, Cu(CN)_3_^2−^ increases gradually, and Cu(CN)_4_^3−^ begins to convert to Cu(CN)_3_^2−^.

**Figure 7:**
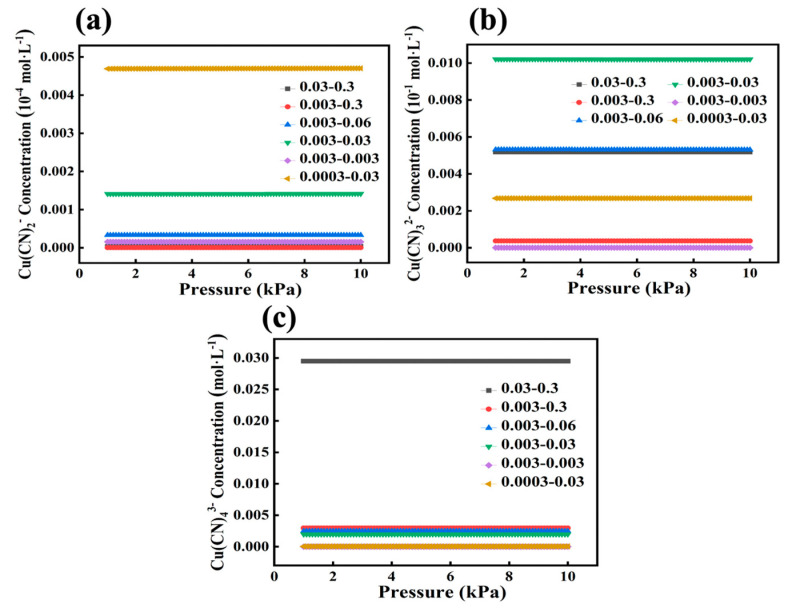
The OLI studio 11.0 simulates the concentration of (**a**) Cu(CN)_2_^−^, (**b**) Cu(CN)_3_^2−^ and (**c**) Cu(CN)_4_^3−^ in the system under different pressures for six different concentration gradients.

Finally, the distribution of three different ionic species with different concentration ratios under different pressure conditions in solution was investigated. As can be seen from Figure 7, the concentrations of the three ionic species do not change with increasing pressure under different concentration gradients, indicating that pressure changes have no effect on the metal cyanide complex ions in the solution state. However, it can still be seen from Figure 7a that at c(Cu):c(CN) = 0.0003:0.003, the amount of Cu(CN)_2_^−^ in the system is the highest among all concentration gradients. It is considered that Cu(CN)_2_^−^ will be the first to be generated in the solution, but since the amount of CN^−^ in the system is too small, to generate Cu(CN)_3_^2−^ and Cu(CN)_4_^3−^, one or two extra CN^−^ are needed compared to generating Cu(CN)_2_^−^. Therefore, under different concentration gradients, the smaller the CN^−^ concentration, the easier it is to generate Cu(CN)_2_^−^. As shown in Figure 7b, the amount of Cu(CN)_3_^2−^ in the system is highest at c(Cu):c(CN) = 0.003:0.3. We believe that due to the large difference in concentration between the metal and CN^−^, with a concentration ratio of 1:100, excess CN^−^ leads to a natural tendency for the system to form the more stable Cu(CN)_3_^2−^. As shown in Figure 7c, the concentration of Cu(CN)_4_^3−^ was the highest among all concentration gradients when c(Cu):c(CN) = 0.03:0.3.

### 2.6. Analysis of the Optimal Process Parameters of Cu(CN)_4_^3−^ Using Response Surface Methodology

In order to further study the impact of the interaction between variables, explore the optimal process parameter range of Cu(CN)_4_^3−^, and screen the optimal process parameter range favorable for precipitation (CuCN), the response surface analysis method was used to select the best Cu(CN)_4_^3−^ processing technology. Based on the BBD sampling principle, three factors that affect Cu(CN)_4_^3−^ were selected for the three-factor, three-level response surface analysis experiment: A (pH), B (temperature), C (c_Cu_:c_CN_). A total of 17 test samples were conducted, with 5 in the central group. The levels of response surface test factors and results can be seen in Tables S3 and S4. The statistical analysis of the experimental data was performed using Design-Expert 10.0.1 software, resulting in a second-order polynomial regression Equation (8). The response mathematical model of Cu(CN)_4_^3−^ was subjected to variance statistical analysis using the Design-Expert 10.0.1 software. Equation (8) was obtained through the analysis of experimental data, as shown in Table S5. The size of the F-value is an important indicator for evaluating the impact of each variable on the response value, where a larger F-value implies a higher contribution of the relevant model component to the response. When the significance test’s probability is *p* < 0.05, it reveals that the variable has a significant impact on the response value and has mathematical statistical significance. From Tables S1–S3, the different processing parameters affecting Cu(CN)_4_^3−^ are sorted by contribution size as follows: C > A > B, i.e., c_Cu_:c_CN_ > pH > temperature and c_Cu_:c_CN_ has a significant impact on Cu(CN)_4_^3−^. The model term is significant, the non-fitting term is not significant, the coefficient of determination R^2^ is 0.9662, and the adjusted coefficient of determination R^2^_adj_ = 0.9228, which can explain 92.28% of the response variability. The high goodness of fit of the model implies that it can be used to navigate the design space, analyze and predict the optimal processing conditions of Cu(CN)_4_^3−^:

(8) $$Y=75.6366+1.23427\times A+0.411833\times B+12.1694\times C-12.4895\times \mathrm{AB}+9.69916\times \mathrm{AC}\;-3.58013\times \mathrm{BC}-34.1696\times A^{2}-20.1627\times B^{2}-22.5539\times C^{2}$$

**Figure 8:**
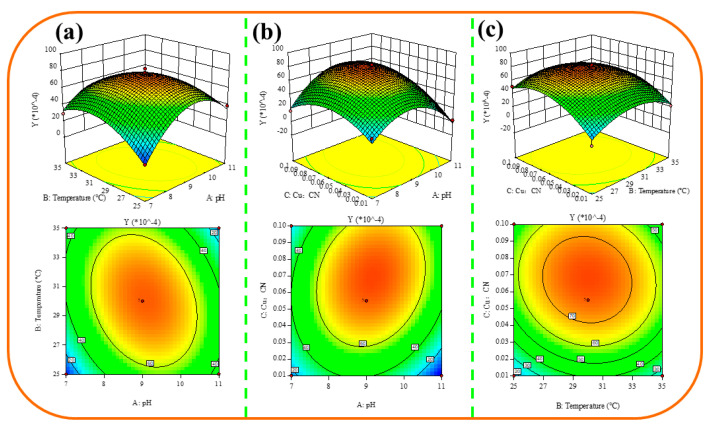
(**a**) Effect of pH and temperature interaction on Cu(CN)_4_^3−^. (**b**) Effect of interaction between pH and c_Cu_:c_CN_ on Cu(CN)_4_^3−^. (**c**) Effect of interaction between temperature and c_Cu_:c_CN_ on Cu(CN)_4_^3−^.

As shown in Figure 8a, the effect of pH-temperature interaction on Cu(CN)_4_^3−^ is analyzed by a parabolic surface, with a large vertical span of the surface and prominent elliptical contour lines of isopleths, indicating a significant interaction between the two factors. When pH is constant, Cu(CN)_4_^3−^ shows an increasing–decreasing trend with increasing temperature. Likewise, when the temperature is constant, Cu(CN)_4_^3−^ shows a trend of first increasing and then decreasing with increasing pH. The optimal process combination, considering only the interaction of the two factors, is near the combination of pH 8–10 and temperature 29–31 °C. As shown in Figure 8b, Cu(CN)_4_^3−^ shows a trend of first increasing and then decreasing with the increase in pH and c_Cu_:c_CN_. At a moderate level (pH of 8–10 and c_Cu_:c_CN_ of 0.05–0.09), the surface shows the highest point, and it is expected that Cu(CN)_4_^3−^ is the highest. The contour lines of the interaction between the two factors show a significant elliptical shape, indicating a significant impact on Cu(CN)_4_^3−^ and is a sensitive impact factor for Cu(CN)_4_^3−^. As shown in Figure 8c, the contour lines of the temperature and c_Cu_:c_CN_ interaction are near circular, and their impact on Cu(CN)_4_^3−^ is not significant. Cu(CN)_4_^3−^ increases first and then decreases with the increase in temperature and c_Cu_:c_CN_, and the highest Cu(CN)_4_^3−^ is obtained when the temperature is in the range of 29–31 °C and c_Cu_:c_CN_ is in the range of 0.06–0.08, which should be controlled to obtain optimized process parameters.

To further accurately determine the global optimal solution, with Y being the maximum as the optimization goal, according to the results obtained from running the Design-Expert 10.0.1 software, the optimal process under the joint influence of pH, temperature, and c_Cu_:c_CN_ is found to be pH = 9.130, temperature = 29.824 °C and c_Cu_:c_CN_ = 0.068, with the model predicting a Cu(CN)_4_^3−^ of 7.7414 × 10^−3^ under these conditions. Based on the results of the response surface analysis, we can conclude that, by determining the values of two relevant variables, we can determine the value of the last variable that leads to the maximum value of Cu(CN)_4_^3−^ in the system. This conclusion provides the optimal pH, temperature, and c_Cu_:c_CN_ process parameter ranges for maximizing Cu(CN)_4_^3−^ in different conditions, thereby determining the optimal process parameters for maximizing CN^−^ deep removal from the solution. This is because, as previously concluded in our study, Cu(CN)_4_^3−^ is the dominant ion form in the complex ion that undergoes precipitation transformation, and only by undergoing transformation to precipitation through Cu(CN)_4_^3−^ can the maximum depth removal of CN^−^ in the solution be realized.

## 3. Materials and Methods

### 3.1. Computational Details

All quantitative calculations were performed using DFT methods. Gaussian16 software was used for structural optimization and thermodynamic calculations [42]. The PBE0-GD3/def2svp theoretical level was selected for optimization. PBE0-GD3/def2svp contains 25% HF and is suitable for transition metal and main group systems [43]. The single-point energies of different spin multiplicities are compared. All structural optimization and thermodynamic calculations are performed without symmetry constraints, relativistic effects, and frozen core treatments. The structures were optimized in both gas and aqueous phases, and a comparison of these structures showed little effect on the solvent environment. Then, the whole optimization was carried out in the gas phase, the frequency corresponding to the optimized structure was calculated and the position was determined to be the minimum. M062x/def2tzvp was used to calculate thermodynamic energies and includes the empirical dispersion correction D3BJ to describe dispersion effects [44,45,46]. An implicit water solvation model is used to simulate the solvent environment [47]. All energy calculations include thermodynamic corrections. Multiwfn is used for the analysis of wave functions [48], electrostatic potential (ESP), and interaction region index (IRI) [49]. Atoms in Molecules (AIM) Electron density at critical points was used for the analysis of bonds, the Laplacian of electron density, the energy and density, and the properties of metal–ligand chemical bonds [50].

### 3.2. Simulation Details

OLI studio 11.0 from OLI Systems, Inc. (Cedar Knolls, NJ, USA). It can predicts multipoint equilibrium calculations for multi-component systems under a variety of operating conditions based on first principles, providing insight into electrolyte and non-electrolyte trends to improve operational efficiency, sustainability, and productivity. Moreover, virtual chemistry laboratory simulation can reduce the experimental cost and speed up the design speed and design the best operation window to improve operation performance and sustainability. The reaction process in the actual system was simulated using the simulation software OLI Studio 11.0 and its accompanying aqueous solution chemical database.

We obtained the results of the detection of the discharge wastewater from a sodium ion battery company after one treatment (Table 1). The results indicated that a significant amount of free cyanide and heavy metal ions still existed in the wastewater after the treatment. Thus, we determined copper (Cu) with the highest concentration in the solution as the research object and studied the complexation between Cu and CN^−^, as well as the transformation path and mechanism of the ion species of the metal cyanide complex formed.

## 4. Conclusions

This study investigated the ionic structure of metal–cyanide complexes formed by the combination of Cu and CN^−^ in discharge wastewater of sodium ion batteries. The transformation laws, transformation paths, complexation mechanisms, and distribution forms of each complex ion were explored. The results showed that Cu exists in the form of Cu(Ⅰ) in the copper cyanide system and only exists in four coordination cases. Based on the bonding relationship between metal ion Cu and ligand CN, the system is more inclined to form Cu(CN)_3_^2−^ spontaneously, and it is more stable and abundant in the solution. The reactions between each complex ion and the formation of precipitation were studied at the quantum chemical level. Cu(CN)_4_^3−^ has a high precipitation property second only to free CN^−^. It was confirmed that Cu(CN)_4_^3−^ is the dominant superior ionic form leading to deep removal. OLI simulation was used to study the transformation path and distribution form of the system under actual trace conditions. The results showed that conventional parameters such as pH, temperature, and pressure can be adjusted to achieve the transformation of CN^−^ in the system from Cu(CN)_2_^−^ to Cu(CN)_3_^2−^ to Cu(CN)_4_^3−^ and finally to form CuCN precipitation, thereby achieving deep removal of cyanide in the solution. The optimum process parameters of Cu(CN)_4_^3−^ under different conditions were determined by response surface analysis, and the optimum process parameters were determined to maximize the depth of CN^−^ removal from the solution. This study provides theoretical support for the development of an eco-friendly and low-cost removal process for wastewater containing cyanide from sodium-ion battery production.

## Figures and Tables

**Table 1:** Detection results for discharge wastewater from a sodium ion battery of a new energy battery company after one treatment.

Serial Number	Test Items	Test Results	Unit
1	As	12.8	μg/L
2	Cd	0.10	μg/L
3	COD	5.10	μg/L
4	Cr	34.9	μg/L
5	Cr^6+^		μg/L
6	Cu	37.0	μg/L
7	Hg	28.7	μg/L
8	Mn		μg/L
9	Ni	32.4	μg/L
10	Pb	6.72	μg/L
11	Ph value	8.69	μg/L
12	Ti	0.19	μg/L
13	Zn	8.93	μg/L
14	cyanide	281.12	μg/L

## Data Availability

The datasets used and/or analysed during the current study available from the corresponding author on reasonable request.

## Supplementary Materials

The following supporting information can be downloaded at: https://www.mdpi.com/article/10.3390/ijms24065066/s1.
