# Supplementary material for: Study on Removal Mechanism for Copper Cyanide Complex Ions in Water: Ion Species Differences and Evolution Process
Source: Int J Mol Sci. 2023 Mar 7;24(6):5066. doi: 10.3390/ijms24065066 (PMC10048877; doi:10.3390/ijms24065066)
Supplement: Supplementary file 1 [file ijms-24-05066-s001.zip › Supplementary Materials.pdf]

Table S1. Optimized bond length of $Cu(CN)_2^-$, $Cu(CN)_3^{2-}$, $Cu(CN)_4^{3-}$ in Cu and CN

Ion	Ions-CN-1	Ions-CN-2	Ions-CN-3	Ions-CN-4	Ions-Charge (opt)	Ions-Charge (sp)
$Cu(CN)_2^-$	1.87194	1.87191			-0.135	0.326
$Cu(CN)_3^{2-}$	1.94263	1.94263	1.94445		-0.537	0.363
$Cu(CN)_4^{3-}$	1.99468	1.99654	1.9947	1.9971	-1.02	0.331

Table S2. Thermodynamic data of $Cu(CN)_2^-$, $Cu(CN)_3^{2-}$, $Cu(CN)_4^{3-}$

Ion	E/a.u.	U/a.u.	H/a.u.	G/a.u.	ΔU/kJ/mol	ΔH/kJ/mol
$Cu(CN)_2^-$	-1826.38	-1826.36	-1826.35	1826.38	54.092	56.571
$Cu(CN)_3^{2-}$	-1919.35	-1919.32	-1919.32	-1919.36	79.618	82.097
$Cu(CN)_4^{3-}$	-2012.31	-2012.27	-2012.17	-2012.32	105.497	105.976

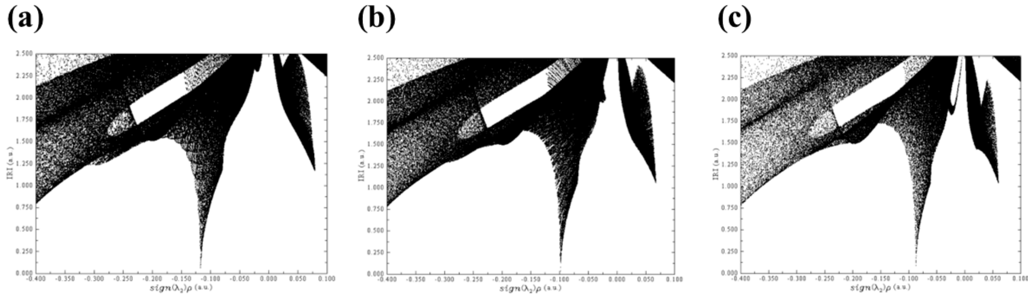

Figure S1. (a)$Cu(CN)_2^-$, (b)$Cu(CN)_3^{2-}$, (c)$Cu(CN)_4^{3-}$ original scatter plot after IRI analysis

Table S3. Response surface test factor level and coding

coding	pH<br>A	Temperature<br>B/°C	c_{Cu}：c_{CN}<br>C
-1	7	25	0.01
0	9	30	0.055
1	11	35	0.1

Table S4. Experimental design and results

Serial number	pH<br>A	Temperature<br>B/℃	c_{Cu}：c_{CN}<br>C	$Cu(CN)_4^{3-}$<br>Y/（*10^-4)
1	1	0	1	35.25034
2	0	-1	1	51.0261
3	0	0	0	74.0659
4	0	0	0	68.2635
5	-1	0	1	16.09322
6	1	1	0	9.17344
7	-1	-1	0	8.45619
8	1	-1	0	38.6135
9	0	-1	-1	8.70559
10	1	0	-1	2.3347
11	0	0	0	82.7417
12	0	0	0	78.1377
13	-1	1	0	28.9742
14	-1	0	-1	21.9742
15	0	1	1	49.9742
16	0	1	-1	21.9742
17	0	0	0	74.9742

Table S5. Results of variance analysis of $Cu(CN)_4^{3-}$ regression equation

Source of variance	Sum of squares	Degree of freedom	variance	F value	P value	significance
Regression model	11971.84	9	1330.20	22.24	0.0002	**
A	12.19	1	12.19	0.20	0.6654	
B	1.36	1	1.36	0.023	0.8845	

C	1184.75	1	1184.75	19.80	0.0030	**
AB	623.95	1	623.95	10.43	0.0145	*
AC	376.29	1	376.29	6.29	0.0405	*
BC	51.27	1	51.27	0.86	0.3854	
A^2	4916.05	1	4916.05	82.18	< 0.0001	**
B^2	1711.72	1	1711.72	28.61	0.0011	**
C^2	2141.80	1	2141.80	35.80	0.0006	**
Residual error	418.76	7	59.82			
Disfitting term	304.75	3	101.58	3.56	0.1256	
Pure error	114.01	4	28.50			
total	12390.60	16				
